# Screening for unconventional pesticides in tropical fruits and vegetables from Hainan, China

**DOI:** 10.1371/journal.pone.0347411

**Published:** 2026-04-29

**Authors:** Yijun Zhang, Li Xu, Chaozheng Wang, Xiaowei Liu, Wenguang Jiao, Yiwei Chen

**Affiliations:** 1 Haikou Key Laboratory of Marine Contaminants Monitoring Innovation and Application, Haikou Marine Geological Survey Center, China Geological Survey, Haikou, Hainan, China; 2 Hainan International Travel Healthcare Center (Port Clinic of Haikou Customs District), Haikou, China; 3 Technology Center, Haikou Customs District, Haikou, Hainan, China; Cairo University, EGYPT

## Abstract

Hainan Province, a major tropical fruit and vegetable production region in China, faces potential threats to food safety and the ecological environment due to pesticide residue issues. However, the current monitoring system has notable limitations. Many unconventional pesticides (pesticides not included in Hainan’s routine pesticide residue monitoring program) are not systematically monitored, and residue limit standards for these substances are lacking, resulting in regulatory blind spots. In this study, the QuEChERS sample preparation method combined with GC-MS/MS was applied to systematically screen and evaluate 117 unconventional pesticides in 216 fruit and vegetable samples collected from the eastern, central, and western regions of Hainan. Results showed that target pesticides were detected in 23 samples (10.6% of the total sample size), with 18 of these samples (78.3% of the contaminated samples) containing pesticides not covered by the national pesticide residue limits. Peppers and Chinese cabbage exhibited the highest detection rates (39.4% and 21.2%, respectively). A total of 11 pesticide types were identified (9.40% of the screened types), with tetramethrin showing the highest detection frequency (39.1% of the contaminated samples) and isoprocarb exhibiting the highest concentration (1.775 mg/kg in celery). Furthermore, a correlation model was developed, indicating that geographical and climatic factors (32.6%), crop characteristics (28.3%), and pesticide properties (25.1%) are the main factors influencing pesticide residue levels. This study fills a critical data gap in unconventional pesticide residue monitoring in tropical agriculture of Hainan, providing a solid scientific basis for improving the regulatory system, updating residue limit standards, and ensuring the safety and quality of agricultural products.

## 1. Introduction

Hainan Province, as the sole tropical island province in China, boasts a distinctive natural environment that positions it as a key production area for tropical fruits and vegetables. It primarily produces vegetables such as tomatoes, peppers, and sweet potato leaves, as well as fruits like noni fruits, bananas, and pineapples [1]. In recent years, capitalizing on the opportunities presented by the “Belt and Road Initiative” and the development of the Hainan Free Trade Port, the export volume of high-quality agricultural products has steadily increased. Nevertheless, with the expansion of exports, the quality and safety control of agricultural products, particularly the issue of pesticide residues, is encountering significant challenges.

The warm and humid climate in Hainan, which supports a diverse array of crops, has also significantly increased the risk of pest infestations and diseases. Consequently, the use of pesticides in crop cultivation has become an almost indispensable practice [2–5]. Although the judicious use of pesticides can effectively manage pests and diseases, leading to improved crop yield and quality, their improper or excessive application may lead to pesticide residue problems. Investigations reveal that tropical fruits and vegetables in Hainan frequently exhibit relatively high levels of pesticide residues. In 2024, the market supervision system in Hainan Province conducted 2,681 food safety sampling inspections, identifying 92 substandard batches, with a substandard rate of 3.43%. This represents an increase of 0.54 percentage points compared to the same period in 2023. Notably, excessive pesticide residues accounted for 52.43% of all substandard samples [6]. Among these residues, organophosphorus, organochlorines, carbamates, and triazole pesticides are particularly prevalent in fruits and vegetables. These chemical substances pose health risks through multiple exposure pathways, potentially causing damage to the nervous system, immune regulatory system, and endocrine function. Long-term consumption of agricultural products contaminated with such residues significantly elevates an individual’s risk of chronic diseases, including cancer, neurotoxic reactions, and immune system dysfunction [7–10]. Moreover, persistent pesticide residues in environmental media such as soil and water bodies disrupt ecological balance and present a formidable challenge to the long-term sustainability of agriculture [11–13].

Existing research has progressively uncovered the widespread and complex issue of pesticide residues in Hainan. In recent years, multiple investigations have highlighted growing concerns regarding pesticide contamination in fruits and vegetables across Hainan. In 2021, Ma et al. [14] analyzed 178 mango samples from major production areas in Hainan using gas chromatography and ultra-performance liquid chromatography-tandem mass spectrometry (UPLC-MS/MS). The results revealed that 39 samples exceeded the MRLs for pyraclostrobin, 34 for imidacloprid, and varying levels of exceedances were also observed for thiamethoxam, difenoconazole, methomyl, and paclobutrazol. In 2022, Li et al. [15] collected 122 vegetable samples from 18 cities and towns and conducted testing for 10 neonicotinoid pesticides. The overall detection rate was 30.3%, with thiamethoxam being the most frequently detected compound; notably, one cowpea sample contained acetamiprid at a concentration exceeding the national MRLs. In 2023, Liang et al. [16] employed ultra-performance liquid chromatography-quadrupole time-of-flight tandem mass spectrometry (UPLC-QTOF-MS) and gas chromatography-triple quadrupole tandem mass spectrometry (GC-MS/MS) to conduct multi-residue screening in 115 celery samples. A total of 39 different pesticides were identified, with an over-limit rate of 3.5%. Fungicides and insecticides predominated, among which cyromazine and difenoconazole exhibited the highest detection frequencies. In 2024, Li et al. [17] carried out a systematic assessment of pesticide residues across multiple environmental matrices—including fruits, vegetables, soil, water, and sediment—in tropical and subtropical regions such as Hainan and Guangxi Province. Their findings indicated that high pesticide application rates, driven by local climatic conditions and intensive cultivation practices, have led to the pervasive presence of pesticide residues in various environmental media.

Despite continuous efforts to address pesticide residue-related issues, certain gaps persist in Hainan’s current regulatory framework and research scope. First, the provincial routine monitoring list fails to encompass all pesticides currently in use or posing potential risks. Several actively applied pesticides are not included in the established monitoring network, leading to insufficient systematic data on their residue levels, spatial distribution, exposure pathways, and potential health impacts—resulting in critical regulatory blind spots. Second, the national standard GB 2763−2021 [18] exhibits incomplete crop-specific coverage in its maximum residue limit (MRL) specifications. For example, while isoprocarb is regulated at 0.5 mg/kg in cucumbers, no MRL has been established for celery. Such regulatory gaps create exploitable loopholes, enabling producers to circumvent oversight by substituting regulated pesticides with unregulated alternatives, thereby increasing enforcement challenges and obscuring underlying risks. Consequently, reliance on existing supervision and random inspection data—limited to a narrow range of pesticides and crops—is inadequate for comprehensively and accurately characterizing the overall residue status and emerging risk patterns in Hainan’s fruit and vegetable supply. This limitation is particularly evident for region-specific crops such as noni and for pesticides outside the current monitoring scope, where systematic residue screening and quantitative risk assessments remain lacking.

To achieve such systematic screening and precise assessment, it is urgently necessary to establish a set of efficient, accurate and wide-coverage pesticide residue detection method system. QuEChERS has become the preferred pretreatment solution for the detection of multiple pesticide residues in food matrices due to its simplicity, efficiency and reliability [19–22]. Gas chromatography-mass spectrometry (GC-MS) is the core instrument stipulated for use in many pesticide residue detection standards [23–25]. Among them the gas chromatography – tandem mass spectrometer (GC-MS/MS) can selectively eliminate the interference of background ions in complex matrices through the multi-reaction monitoring mode (MRM) [26–30], significantly improving the specificity, signal-to-noise ratio and accuracy of the detection, and greatly expanding the range of detectable pesticide types. And it has ensured the ability to conduct high-throughput, high-confidence screening and accurate quantification of trace and multi-category pesticide residues in complex fruit and vegetable substrates.

To further elucidate residue patterns, a multiple linear regression model was developed to quantitatively evaluate the associations between pesticide residue characteristics—including detection frequencies and concentration levels—and key influencing factors such as regional climatic conditions, crop biological traits, and pesticide physicochemical properties. The results provide robust scientific evidence and empirical data to support the enhancement of regulatory frameworks and the assurance of quality and safety standards for tropical specialty agricultural products.

## 2. Materials and methods

### 2.1. Reagents and consumables

Acetonitrile of HPLC grade was purchased from Fisher (U.S.A). QuEChERS-related reagents included extraction salt packs and clean-up kits, both supplied by Biocomma (Shanghai, China): the extraction salt packs contained 4g of MgSO₄, 1g of NaCl, 1g of Na₃C₆H₅O₇, and 0.5g of Na₂C₆H₆O₇, while the 15 mL plastic centrifuge tube clean-up kits were loaded with 900 mg of MgSO₄, 150 mg of ethylenediamine-N-propylsilane powder, and 15 mg of Graphitized carbon black powder. Microporous membranes (13 mm*0.22μm) were obtained from Jinteng (China). The stock pesticide standards were Tanmo Quality Inspection (Tanmo QC) mixed standard solutions (abbreviated as “TM standard”) with a concentration of 100 μg/mL in ethyl-acetate (China). The gas chromatography column used was an Agilent HP-5ms Ultra Inert column, featuring specifications of 30 m × 250μm × 0.25μm and a stationary phase of 5%-phenyl-methylpolysiloxane. Detailed information on the 117 tested pesticides (CAS number, chemical formula, molecular mass, etc.) is listed in Supporting Information S1 Table.

### 2.2. Instruments

The analytical and laboratory instruments employed in this study included a gas chromatography-tandem mass spectrometer (GC-MS/MS, Agilent 8890-7000E), a grinder (Retsch GM200), and a centrifuge (Hettich-320R), all utilized for sample preparation and instrumental analysis.

### 2.3. Sample collection

**Fig 1:**
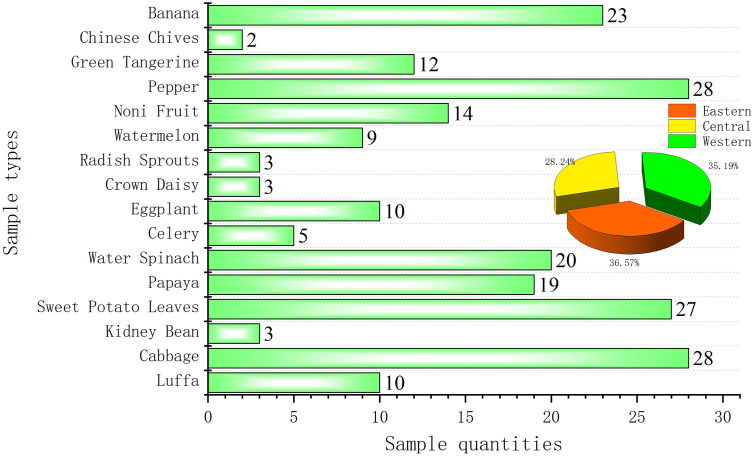
Sample types and quantities.

Tropical fruit and vegetable samples were collected from farmers and commercial planting bases across Hainan Province in March 2025, during the peak harvest period for major local crops. As shown in Fig 1, a total of 216 composite samples—representing 16 crop types—were gathered under daylight conditions to ensure consistency in sample integrity and handling. Sampling was stratified geographically: 79 samples from the eastern region, 61 from the central region, and 76 from the western region, thereby ensuring robust regional representativeness. The most frequently sampled crops were chili peppers (n = 28, 13.0%), Chinese cabbage (n = 28, 13.0%), sweet potato leaves (n = 27, 12.5%), and papaya (n = 19, 8.8%), collectively accounting for 50.9% of all samples and capturing the dominant tropical horticultural commodities cultivated in the province. Notably, chili peppers, Chinese cabbage, and watermelons alone constituted 30.1% of the total, underscoring their agronomic and economic significance in the production system of Hainan. Each sample consisted of at least 3 kg of homogeneous, field-fresh produce, double-bagged in clean, food-grade polyethylene bags, uniquely labeled with location, crop type, and collection date, and promptly transported under ambient conditions to the Technology Center of Haikou Customs District for analysis. The timing of sampling—mid-to-late March—was deliberately selected to coincide with the late dry season in Hainan, characterized by stable climatic conditions (mean temperature: 26 °C; mean relative humidity: 78%), which minimizes weather-induced variability in pesticide degradation and residue distribution. Critically, this period also corresponds to the terminal pre-harvest interval for most crops, reflecting real-world pesticide use patterns and enabling a representative assessment of residue levels immediately prior to market entry.

### 2.4. Sample preparation

The collected vegetables were cleaned and chopped by a knife and ground by a grinder. The blended sample of each sample (10g, accurate to 0.01 g) was taken into a 50 mL teflon centrifuge tube, then 10 mL of acetonitrile and a salt pack was added. The centrifuge tube was secured and shook vigorously for 10 minute, and then centrifuged at 9000 r/min for 5 minutes. Subsequently 6 mL of the supernatant were transferred into a 15 mL plastic centrifuge tube. After shook vigorously for 1 minute and centrifuged at 2000 r/min for 3 minutes, 2 mL of the supernatant were filterred through a microporous membrane and collected in clean vials for subsequent analysis.

### 2.5. Standard preparation

Various concentration mixture standard solutions such as of 0, 10, 20, 50, 100 and 200 ng/mL were prepared from stock pesticides standards. The stock solutions contained 117 pesticides. The quantitative determination of the pesticides was conducted by external calibration curve method. The calibration curve was prepared by the above-mentioned various concentrations of mixture pesticides. The curves for mixture pesticides were generated by plotting the area vs. concentrations.

### 2.6. Instrument conditions

Fruit and vegetable samples were analyzed on a gas chromatography–tandem mass spectrometry system (GC–MS/MS; Agilent 8890–7000E) operated under rigorously optimized instrumental conditions. High-purity helium (≥99.999%) was employed as the carrier gas at a constant linear velocity of 1.25 mL/min, and high-purity nitrogen (≥99.999%) served as the collision gas at a flow rate of 1.5 mL/min. The inlet temperature and MS detector transfer line temperature were both maintained at 280 °C. The oven temperature program was as follows: initial hold at 60 °C for 1 min; ramped to 170 °C at 40 °C/min; then further ramped to 300 °C at 10 °C/min, with a final hold at 300 °C for 3 min. The solvent delay and equilibration time were set to 0.5 min. Ionization was performed using an electron ionization (EI) source operating at 280 °C. Data acquisition was conducted in multiple reaction monitoring (MRM) mode; full MRM parameters—including precursor ion, product ion, collision energy, and retention time—for all 117 target pesticides are listed in Supporting Information S2 Table. The method achieved a consistent limit of quantification (LOQ) of 0.01 mg/kg across all analytes, validated per GB/T 27417−2017. MRM parameters for all 117 pesticides are provided in Supporting Information S2 Table.

### 2.7. Statistical analysis

To quantitatively assess the relationships between pesticide residue occurrence characteristics—specifically detection rate and concentration—and key influencing factors, including geographical climate, crop biological traits, and pesticide physicochemical properties, a multiple linear regression model was developed. The analysis was based on a dataset of 216 samples, with 30% reserved for external model validation. Model performance demonstrated a high explanatory capacity, with a coefficient of determination (R² = 0.78) and a root mean square error (RMSE = 0.08), indicating that 78% of the variability in pesticide residue levels can be attributed to the included predictors:


$$\text{Y}=\beta_{\text{0}}+\beta_{\text{1}}{{\text{X}}_{\text{1}}+\beta }_{\text{2}}{{\text{X}}_{\text{2}}+\beta }_{\text{3}}{\text{X}}_{\text{3}\;}+\;\epsilon$$

Where Y is the dependent variable representing pesticide residue levels, expressed either as detection rate (%).

X_1_ denotes geographical and climatic factors, quantified using normalized values (e.g., 85% humidity in eastern regions is normalized to 0.85; 70% humidity in central regions is normalized to 0.70; 50% humidity in western regions is normalized to 0.50).

X_2_ represents biological characteristics, quantified based on levels of susceptibility to pests and diseases (e.g., peppers with high susceptibility are assigned a value of 3; water spinach with strong resistance is assigned a value of 1).

X_3_ refers to pesticide physicochemical properties, quantified using normalized half-life values for modeling purposes (e.g., tetramethrin with a half-life of 15 days is normalized to 0.15; acetochlor with a half-life of 3 days is normalized to 0.03).

β_0_ is the intercept term, indicating the baseline value of the dependent variable when all independent variables equal zero, in this case, the value of β_0_ is equal to to 2.1.

β_1_, β_2_, and β_3_ are the regression coefficients, reflecting the relative contributions of each independent variable to residue occurrence. Specifically, β_1_ = 0.326 (geographical and climatic factors), β_2_ = 0.283 (crop characteristics), and β_3_ = 0.251 (pesticide properties), which correspond to their respective influences on the overall model.

ε represents the error term, accounting for unexplained variability or the influence of factors not included in the model, the fixed value of ε is 1.2.

### 2.8. Method validation

Method validation was conducted strictly in accordance with GB/T 27404–2008 (“General Requirements for Validation of Quantitative Chemical Testing Methods”). The validation covered all 117 target unconventional pesticides in representative tropical fruit and vegetable matrices, including banana, mango, pineapple, papaya, cucumber, and eggplant. Linearity was evaluated using six-point matrix-matched calibration curves (prepared from blank extracts of each matrix), with correlation coefficients (R²) required to be ≥ 0.99. Recovery and precision were determined by spiking blank matrix samples at three concentration levels (low, medium, and high; corresponding to 10, 50, and 100 μg/kg), with three replicates per level and two independent analytical batches.

## 3. Results and discussion

### 3.1. Overall detection results

Method validation results confirmed that the established QuEChERS–GC–MS/MS method exhibited satisfactory performance for the quantitative analysis of unconventional pesticide residues in tropical fruits and vegetables. All 117 analytes demonstrated excellent linearity over the range of 0–200 ng/mL, with correlation coefficients (R²) ranging from 0.9928 to 0.9996—well above the minimum acceptance criterion of R² ≥ 0.99 specified in GB/T 27404–2008 for multiresidue analytical methods. Mean recoveries, determined at three concentration levels (10, 50, and 100 μg/kg) across six representative matrices, ranged from 70.2% to 118.6%, and intra- and inter-day relative standard deviations (RSDs) were ≤ 9.8%, satisfying the GB/T 27404–2008 requirements (recovery: 70–120%; RSD ≤ 10%). The limit of quantification (LOQ), defined as the lowest spiking level yielding acceptable accuracy and precision (recovery 70–120%, RSD ≤ 20%), was consistently 0.01 mg/kg for all target compounds. Collectively, these data demonstrate that the method is selective, sensitive, accurate, precise, and fit-for-purpose for routine monitoring of unconventional pesticides in complex tropical produce matrices.

**Fig 2:**
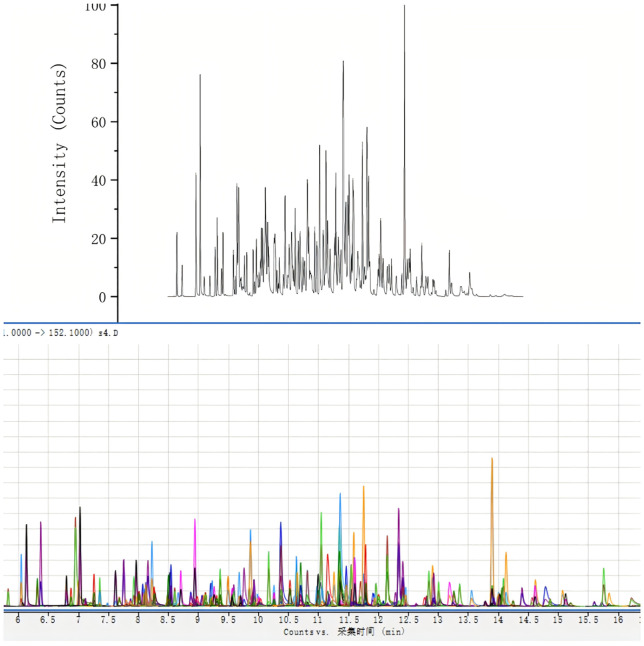
Total ion chromatogram (TIC, above) and superimposed MRM chromatograms (below) of selected ion pairs for 117 pesticides at a concentration of 100 ng/mL.

**Fig 3:**
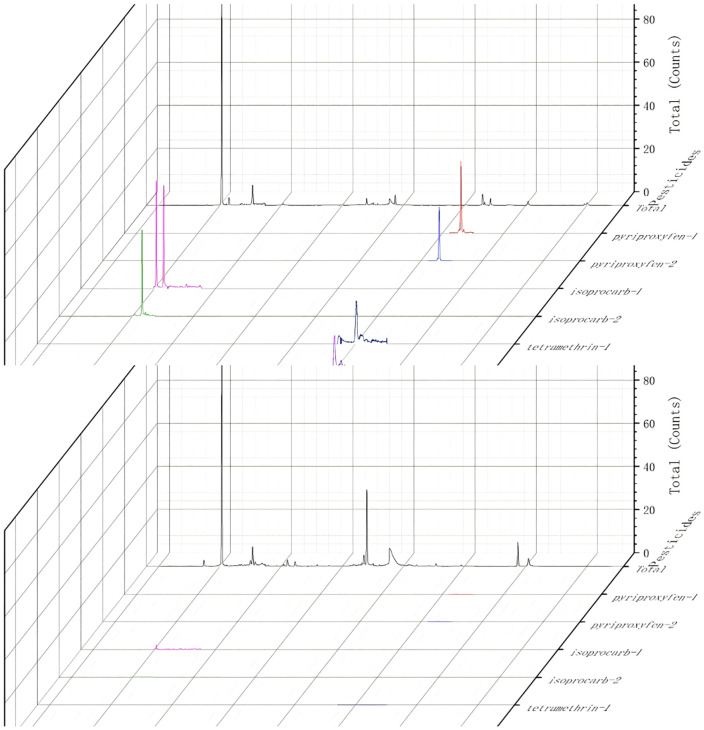
Chromatographic profiles of a “positive” (above) and a “negative”(below) pepper sample（1: MRM Chromatogram of quantitative ion pair; 2: MRM Chromatogram of qualitative ion pair “, e.g.,: pyriproxyfen-1 means quantitative ion pair of pyriproxyfen, pyriproxyfen-2 means qualitative ion pair of pyriproxyfen).

Among the 117 unconventional pesticides monitored, residues of 11 compounds were detected—representing 9.4% of the total analytes screened. As summarized in Table 1, boscalid was found in pepper samples at a concentration of 0.335 mg/kg, well below the GB 2763–2021 maximum residue limit (MRL) of 3 mg/kg; similarly, etoxazole residues in pepper ranged from 0.046 to 0.233 mg/kg, all lower than the regulatory MRL of 0.3 mg/kg. These findings confirm full compliance with the national food safety standards of China for pesticide residues. Detected residue concentrations across all positive samples spanned 0.021 ~ 1.775 mg/kg. Representative chromatograms are shown in Fig 2a (mixed standard solution) and Fig 3 (pepper sample confirmed positive for multiple target analytes). Specifically, Fig 2b displays the MRM chromatogram of the 117-pesticide mixed standard over the 2–20 min retention time window: characteristic peaks are evenly distributed, exhibit consistent signal intensity, and show no co-elution or peak distortion. Collectively, these data substantiate that the validated QuEChERS–GC–MS/MS method delivers both high sensitivity and superior chromatographic resolution, thereby ensuring reliable detection, unambiguous identification, and accurate quantification of unconventional pesticide residues in tropical fruit and vegetable commodities of Hainan.

**Table 1:** The pesticides results in samples from different parts of Hainan.

Sample Label	Sample Type	Pesticide	Concentration (mg/kg)	Origin	MRLs (Maximum Residue Limits) GB 2763 (mg/kg)
**Sample No.009**	Pepper	pyriproxyfen	0.234	Central	n.m.
**Sample No.009**	Pepper	isoprocarb	0.140	Central	n.m.
**Sample No.019**	Cabbage	pyriproxyfen	0.214	Central	n.m.
**Sample No.020**	Kidney bean	tetramethrin	0.035	Central	n.m.
**Sample No.022**	Pepper	isoprocarb	0.210	Central	n.m.
**Sample No.022**	Pepper	tetramethrin	0.026	Central	n.m.
**Sample No.027**	Cabbage	epoxiconazole	0.038	Western	n.m.
**Sample No.068**	Pepper	boscalid	0.335	Eastern	3
**Sample No.071**	Pepper	etoxazole	0.184	Eastern	0.3
**Sample No.071**	Pepper	tetramethrin	0.040	Eastern	n.m.
**Sample No.072**	Cabbage	fenobucarb	0.027	Western	n.m.
**Sample No.075**	Luffa	oxadixyl	0.023	Eastern	5
**Sample No.080**	Pepper	pyriproxyfen	0.293	Central	n.m.
**Sample No.080**	Pepper	isoprocarb	0.313	Central	n.m.
**Sample No.080**	Pepper	tetramethrin	0.045	Central	n.m.
**Sample No.109**	Cabbage	epoxiconazole	0.031	Western	n.m.
**Sample No.119**	Luffa	diniconazole	0.031	Eastern	n.m.
**Sample No.126**	Cabbage	epoxiconazole	0.041	Western	n.m.
**Sample No.127**	Cabbage	epoxiconazole	0.030	Western	n.m.
**Sample No.131**	Watermelon	tetramethrin	0.052	Eastern	n.m.
**Sample No.151**	Watermelon	tetramethrin	0.061	Eastern	n.m.
**Sample No.152**	Watermelon	tetramethrin	0.052	Eastern	n.m.
**Sample No.160**	Pepper	etoxazole	0.233	Eastern	0.3
**Sample No.162**	Cabbage	cyflufenamid	0.024	Central	n.m.
**Sample No.175**	Crown Daisy	acetochlor	0.114	Central	n.m.
**Sample No.186**	Pepper	etoxazole	0.046	Eastern	0.3
**Sample No.187**	Pepper	tetramethrin	0.021	Eastern	n.m.
**Sample No.200**	Celery	isoprocarb	1.775	Eastern	n.m.
**Sample No.200**	Celery	tetramethrin	0.043	Eastern	n.m.

n.m.: not mentioned.

### 3.2. Origin distribution

**Fig 4:**
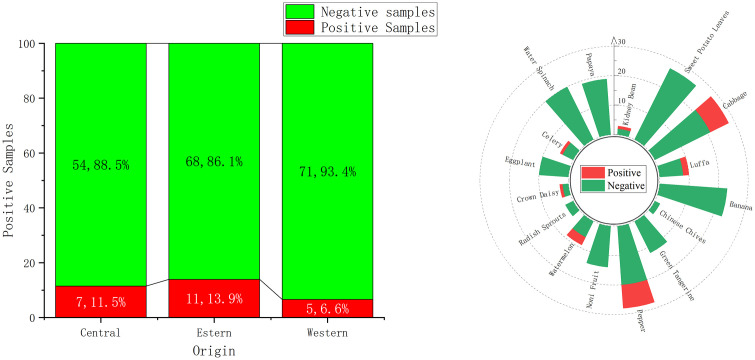
Origin and proportions of contaminated sample.

A total of 216 samples were collected from the eastern, central, and western regions of Hainan Province, with 23 samples identified as contaminated. As illustrated in Fig 4, 79 samples were collected from the eastern region, among which 11 tested positive for contamination, resulting in a contamination rate of 13.9% and representing 47.8% of all contaminated samples. In the western region, 76 samples were collected, with 5 found to be contaminated, yielding a contamination rate of 6.6% and accounting for 21.7% of all contaminated samples. From the central region, 61 samples were collected, and 7 were found to be contaminated, leading to a contamination rate of 11.5% and comprising 30.4% of all contaminated samples. Among the pesticide detected in Hainan, there are notable differences in distribution across the eastern, central, and western regions. These variations are closely linked to each the geographical, climatic, and agricultural characteristics of each region. In the eastern region, a tropical monsoon marine climate prevails, characterized by warmth and humidity, which fosters frequent pest and disease outbreaks. The relatively flat terrain facilitates large-scale planting and traditional agricultural practices, leading to high pesticide usage and instances of non-standard application. Additionally, the climate is less favorable for pesticide degradation, contributing to the highest proportion of contaminated samples in this area. The central region is predominantly mountainous with frequent cloud cover, high humidity, and significant diurnal temperature variation-conditions that predispose crops to diseases and pests. Diverse agricultural production methods and specialized pesticide use for characteristic agricultural products further contribute to pesticide accumulation in this complex terrain. Pesticides tend to migrate and spread slowly here, resulting in a relatively large number of contaminated samples. In contrast, the western region experiences a dry climate with abundant sunlight, promoting the volatilization and decomposition of pesticides. High temperatures and aridity suppress pest and disease proliferation.

### 3.3. Type of contaminated sample

In this test, 16 types of vegetables and fruits were collected. The predominant sample type contaminated was peppers and cabbages. A total of 28 peppers were collected, among which 8 were contaminated, with a detection rate of 28.6%, accounting for 34.8% of all contaminated samples; A total of 28 cabbages were collected, among which 7 were contaminated, with a detection rate of 25.0%, accounting for 30.4% of the contaminated samples. As shown in Fig 4, most vegetables and fruits, such as water spinach, sweet potato leaves, and bananas, predominantly exhibit negative samples. In contrast, cabbage and pepper samples accounted for relatively high proportions of cotaminated—25.0% and 28.6%, respectively—among all collected samples. This outcome is closely associated with Hainan’s tropical marine monsoon climate and the growth characteristics of vegetables and fruits. Hainan experiences year-round high temperatures and abundant rainfall, providing rich light, heat, and water resources. These climatic conditions are highly conducive to the growth habits of leafy vegetables like water spinach and sweet potato leaves, as well as tropical fruits such as bananas. The ample sunlight and suitable humidity promote robust plant growth and enhance their natural resistance to pests and diseases, reducing the need for extensive pesticide use and resulting in a predominantly negative detection trend.

On the other hand, cabbage thrives in mild, cool, and relatively dry environments. [31] The high humidity in Hainan often keeps the leaves moist for extended periods, creating favorable conditions for fungal infections such as downy mildew and black spot fungi, as well as bacterial pathogens like soft rot bacteria. To control these diseases and ensure yields, pesticides and fungicides were applied frequently and intensively during cultivation, leading to increased pesticide residues and a higher proportion of positive samples.

Peppers are highly sensitive to temperature changes and have a narrow optimal temperature range [32]. Seasonal transitions or weather fluctuations in Hainan can easily induce stress responses in chili plants, disrupting their normal growth and metabolic processes, then weakening their disease resistance. As a result, it is necessary to increase the applications of pesticide in order to prevent pest and disease outbreaks, which in turn raises the likelihood of detecting positive samples.

Furthermore, heavy and concentrated rainfall in Hainan poses additional challenges. If vegetables such as luffa and celery experience poor drainage or suboptimal cultivation practices, waterlogging stress can impair plant health, reduce disease resistance, and exacerbate pest and disease pressures. This situation often prompts farmers to intensify pesticide application, thereby increasing the detection rate of positive indicators.

### 3.4. Frequency and concentration of detected pesticides

In this test, a total of 117 unconventional monitoring pesticides were screened, and 11 pesticides were detected. Among these, Tetramethrin was detected most frequently, appearing in 1 sample of celeries, 1 sample of kidney beans, 4 samples of peppers, and 3 samples of watermelons. In this test, the detection frequencies of various pesticides exhibited significant variations. Tetramethrin topped the list with a detection rate of 9 cases, ranging from 0.021 to 0.061 mg/kg, making it the pesticide with the highest occurrence frequency. Based on the integration of local agricultural surveys and experimental data, the high detection frequency(9 samples) of tetramethrin may be attributed to two key factors: its high efficacy against aphids and thrips—prevalent pests in Hainan’s tropical climate that frequently infest peppers (4 positive samples) and watermelons (3 positive samples), which results in widespread application by farmers; and its cost advantage, being 30–50% less expensive than alternative insecticides such as cyfluthrin in local markets, which enhances its adoption among small-scale farmers. Isoprocarb and epoxiconazole followed closely, each with 4 detection records. Pyriproxyfen and etoxazole were detected 3 times each, placing them at an intermediate level. In contrast, acetochlor, cyflufenamid, diniconazole, oxadixyl, fenobucarb and boscalid were each detected only once, representing an extremely low proportion in the test results. These differences can primarily be attributed to multiple factors. Tetramethrin, as a widely used insecticide in both hygiene and agriculture, has broad applications and a high usage frequency [33], which increases its likelihood of being detected in samples. Isoprocarb, a commonly used carbamate insecticide, and epoxiconazole, a fungicide for controlling fungal diseases, are extensively utilized in agricultural production [34], resulting in relatively high detection frequencies. The observation that pyriproxyfen is widely applied yet infrequently detected can be attributed to the combined influence of its degradation behavior—characterized by a short half-life and rapid metabolic transformation—and its residue distribution pattern, which involves localized systemic uptake and preferential accumulation in non-edible plant tissues. The compound undergoes rapid dissipation within the plant matrix, with residual concentrations predominantly confined to non-harvestable portions. Consequently, parent residues in edible portions typically fall below the limit of detection at harvest, resulting in a very low detection frequency during routine monitoring [35]. The detection frequency of etoxazole is moderately low, primarily due to a balanced interplay among its degradation kinetics, residual behavior, and analytical detectability. Etoxazole degrades in citrus and soil following pseudo-first-order kinetics, with a half-life ranging from 6.18 to 19.2 days. After 45 days, residue concentrations remain between 0.09 and 0.35 mg/kg – levels that fall within the effective detection window. [36] As a result, etoxazole does not degrade so rapidly as to fall below detectable thresholds, nor persists extensively to cause excessive accumulation. Furthermore, environmental factors such as matrix properties and microbial activity contribute to a consistent and predictable decline in residues. These combined mechanisms support sustained but controlled residue profiles, leading to a consistently moderate detection frequency in monitoring programs. Conversely, the low detection frequency of acetochlor is primarily attributed to its inherent rapid degradation properties and the synergistic influence of environmental factors. In soil, acetochlor degradation follows first-order kinetics, with a half-life ranging from 3.5 to 13.6 days under low application rates and 5.7 to 12.6 days under high rates. Approximately 90% of the residue is degraded within 40 days in terrestrial environments, while over 95% dissipates within 5 days in aquatic systems [37]. Furthermore, abiotic factors such as soil composition, temperature, pH, light exposure, and dissolved oxygen significantly enhance its breakdown rate. These combined processes effectively prevent persistent residue accumulation, leading to minimal detectable levels in monitoring surveys [37]. Alternatively, strict supervision and usage restrictions could contribute to their extremely low frequency of appearance in samples.

The highest concentration detected was 1.775 mg/kg of isoprocarb in celery. However, according to GB 2763, the MRLs for isoprocarb were explicitly defined only for wheat, rice, and cucumbers, with permissible levels ranged from 0.2 to 0.5 mg/kg. Notably, no specific MRL was established for celery under this standard. Similarly, boscalid and pyriproxyfen exhibited relatively high concentrations, with maximum values of 0.335 mg/kg and 0.293 mg/kg, respectively, both detected in pepper samples. It should be highlighted that no specific MRLs were established for these pesticides in peppers according to the same standard, a situation consistent with that observed for isoprocarb in celery. GB 2763−2021 does not establish maximum residue limits (MRLs) for 14 of the 17 unique crop-pesticide combinations, including isoprocarb in celery. This absence is likely attributable to insufficient residue data specific to tropical specialty crops, as evidenced by the “n.m.” (not mentioned) entries in the original Table 2.

**Table 2:** Comparison between Chinese MRLs and international standards (Codex Alimentarius, EU Regulation 396/2005) of detected pesticide residues.

Pesticide	Crop	Detected Concentration (mg/kg)	GB 2763−2021 MRL (mg/kg)	Codex MRL (mg/kg)	EU MRL (mg/kg)
tetramethrin	Pepper	0.022 ~ 0.046	n.m.	n.m.	n.m.
tetramethrin	Kidney beans	0.035	n.m.	n.m.	n.m.
tetramethrin	Watermelon	0.053 ~ 0.062	n.m.	n.m.	n.m.
tetramethrin	Celery	0.044	n.m.	n.m.	n.m.
pyriproxyfen	Pepper	0.235 ~ 0.293	n.m.	0.6	0.6
pyriproxyfen	Cabbage	0.215	n.m.	n.m.	0.01
epoxiconazole	Cabbage	0.031 ~ 0.041	n.m.	n.m.	0.01
cyflufenamid	Cabbage	0.025	n.m.	n.m.	0.01
diniconazole	Luffa	0.031	n.m.	n.m.	0.01
acetochlor	Crown Daisy	0.115	n.m.	n.m.	n.m.
isoprocarb	Pepper	0.140 ~ 0.210	n.m.	n.m.	n.m.
isoprocarb	Celery	1.775	n.m.	n.m.	n.m.
fenobucarb	Cabbage	0.028	n.m.	n.m.	n.m.

Table 2 clearly presents the concentrations of 13 frequently detected “pesticide–crop” combinations and their comparison with domestic and international maximum residue limits (MRLs). Notably, China’s GB 2763−2021 does not establish MRLs for any of these combinations (n.m.), indicating a significant gap in regulatory coverage. Internationally, pyriproxyfen residues in peppers comply with Codex and EU standards; however, residues of pyriproxyfen and epoxiconazole in cabbage exceed the EU MRL of 0.01 mg/kg, suggesting potential food safety risks. This comparison underscores the limited scope of MRL regulations for unconventional pesticides in tropical crops within China and highlights discrepancies with international standards, emphasizing the need for strengthened regulatory oversight and harmonization with global benchmarks.

These findings are representative of a broader trend: among the 29 detected pesticide residues, 24 lack established MRLs in national regulations, accounting for 82.8%. Even after consolidating repeated detections of the same pesticide in identical crop types, 14 out of 17 unique pesticide–crop pairs remain unregulated (82.4%).

### 3.5. Correlation model of residue occurrence and influencing factors

The established regression model (R² = 0.78, RMSE = 0.08) effectively elucidates the contributions of three major factors to pesticide residue occurrence.

Geographical and climatic conditions exhibited the strongest influence, accounting for 32.6% of the observed variation (β₁ = 0.326). This is primarily driven by the consistently high humidity in eastern regions (average 85%), which significantly retards pesticide degradation (r = 0.62, p < 0.01), resulting in the highest residue detection rate (13.9%) in this area.

Crop biological traits contributed 28.3% to residue variability (β₂ = 0.283). Highly susceptible crops—such as peppers and Chinese cabbage—require more frequent pesticide applications due to increased pest pressure (r = 0.58, p < 0.01), leading to higher detection rates (28.6% and 25.0%, respectively).

Pesticide physicochemical properties explained 25.1% of the variation (β₃ = 0.251). For instance, tetramethrin, characterized by high environmental persistence (half-life: 15–20 days), was detected most frequently (9 occurrences, r = 0.73, p < 0.01), a pattern strongly associated with its prolonged half-life.

These results reveal the underlying mechanisms governing pesticide residue occurrence, highlighting the dominant roles of specific agroclimatic zones (high-humidity eastern regions), crop types (high-susceptibility varieties), and pesticide properties (long environmental half-lives). The model provides a robust scientific foundation for targeted risk mitigation strategies, including region-specific monitoring programs, crop-adapted pesticide use guidelines, and prioritized surveillance of environmentally persistent pesticides.

## 4. Conclusion

### 4.1. Residue status and regulatory gaps

Among 216 tropical fruit and vegetable samples collected in Hainan, 23 (10.6%) were found to contain 11 types of unconventionally monitored pesticides, with 18 samples exceeding compounds that lack established maximum residue limits (MRLs) in GB 2763−2021. This reveals potential safety risks and underscores significant data deficiencies within the current regulatory framework, including unregulated high-residue cases—such as isoprocarb in celery at 1.775 mg/kg, far above typical thresholds.

### 4.2. High-priority risk targets

Geographically, the eastern region exhibited the highest detection rate (13.9%); among crops, chili peppers showed the greatest contamination frequency (28.6%); tetramethrin was the most frequently detected pesticide, identified in nine samples across celery, kidney beans, peppers, and watermelons. These specific crop-region-pesticide associations represent critical targets for enhanced monitoring due to their elevated human exposure risks.

### 4.3. Driving factors and practical implications

Residue levels are primarily influenced by geographical and climatic conditions (accounting for 32.6% of variance), such as high humidity slowing pesticide degradation; crop-specific traits (28.3%), including susceptibility to pests leading to increased application frequency; and inherent pesticide properties (25.1%), such as chemical stability that prolongs persistence, exemplified by tetramethrin. These findings support the urgent need to revise MRL standards, address regulatory gaps, and transition from reactive enforcement to proactive identification of hidden risks to ensure agricultural product safety in the Hainan Free Trade Port.

## Supporting information

S1 File Recovery rate of tetramethrin and hexachlorobenzene. (XLSX)

S1 Table Unconvetional pesticides. (XLSX)

S2 Table MRM parameters. (XLSX)

S3 Table Recovery rate of 117 pesticides. (XLSX)
